# Nature versus nurture: genetic background and media composition shape endothelial cell transcriptomes in vitro

**DOI:** 10.1038/s41598-026-43732-0

**Published:** 2026-03-16

**Authors:** Flóra Demeter, Márta Lídia Debreczeni, Zsuzsanna Németh, Erika Kajdácsi, Zoltán Doleschall, László Cervenak

**Affiliations:** 1 Department of Internal Medicine and Hematology, Semmelweis University, Szentkirályi u. 46, Budapest, 1088 Hungary; 2 Research Group for Immunology and Hematology, Semmelweis University-HUN-REN-SU (Office for Supported Research Groups), Budapest, Hungary; 3 Department of Pathogenetics, National Institute of Oncology, Budapest, Hungary

**Keywords:** Transcriptomics, Genetic heterogeneity, Cell culture media, Primary cells, In vitro, Human umbilical vein endothelial cell, Cell biology, Genetics, Molecular biology

## Abstract

Primary endothelial cells (e.g., HUVEC) are essential in basic research and drug development, yet contradictory or poorly reproducible experimental results are common in the literature. We investigated whether such discrepancies arise from differences in culture media or donor genetic backgrounds using Agilent microarray transcriptomic analysis of three donor-derived HUVEC lines cultured in four distinct media (regarding basic composition, serum- and growth factor content). Genome-wide cluster analysis of 13,823 expressed genes revealed that samples clustered primarily by medium. Of the 2215 genes showing differential expression, 1697 varied across media, while only 805 were affected by donor differences. Correlation and principal component analysis confirmed that media composition had a strong impact on gene expression. Functional analysis of nine key endothelial functions showed predominantly medium-dependent effects, particularly on mitosis, angiogenesis, inflammation, and adhesion. The pivotal role of media composition in shaping HUVEC transcriptome may underlie inconsistencies across studies.

**Supplementary Information:**

The online version contains supplementary material available at 10.1038/s41598-026-43732-0.

## Introduction

The significance of endothelial cells (ECs) is self-evident: forming the innermost layer of vessel walls, they are ubiquitous throughout the body and regulate fundamental physiological processes, such as blood pressure, transport of metabolites, homing of leukocytes, etc. A deep understanding of endothelial cell biology is crucial for both uncovering the mechanisms behind their functions and evaluating the efficacy and safety of diverse pharmaceuticals that enter the bloodstream. Investigating the multifaceted mechanisms of endothelial cell function should initially be undertaken in simplified experimental model systems, for which primary human umbilical vein endothelial cells (HUVECs) present a remarkably advantageous tool. HUVECs are isolated from fresh umbilical cords of healthy neonates, which makes these cells relatively easily and predictably accessible. Due to their neonatal origin, they are also less influenced by environmental and lifestyle factors. These advantages have made HUVECs a widely used in vitro endothelial cell model: a PubMed search yields over 306,000 results for “endothelial cell”, and more than 39,000 when combined with the term "HUVEC” or “human umbilical vein endothelial cells”.

In our laboratory, we have dedicated the past two decades to investigating HUVEC cells in 2D in vitro cultures. During this time, we have observed a degree of variability in the response patterns of these primary endothelial cells, in line with reports from other research groups. Moreover, we have frequently encountered contradictory findings, both in literature and in our own experiments. Notable examples include inconsistent results related to endothelial permeability^1,2^, cell migration/angiogenesis^3,4^, and endothelial lactate production^5,6^. Such inconsistencies are part of the broader reproducibility crisis, well-documented in biomedical research, where reproducibility rates have been estimated to be as low as 15%^7^. Although the causes are multifactorial, identifying each contributing variable is essential to address one of the most concerning issues of today’s science.

In the case of primary cells like HUVECs, two major sources of variability can be the composition of the cell culture media and the genetic background of the donor. Within our own studies, we have repeatedly observed that the behavior of endothelial cells in vitro is strongly influenced by the composition of the culture medium. Therefore, we always begin by optimizing the culture medium for each experimental system. Despite the extensive scientific literature, there is no universally accepted consensus regarding the optimal composition of culture media for in vitro culturing and specific assays, leading to the widespread use of various formulations. In our group, for instance, we routinely use four types of media when working with endothelial cells, each optimized for a different purpose. For HUVEC isolation and general culturing, we use ‘MCDB’ (an MCDB-131-based medium supplemented with 5% fetal bovine serum and multiple growth factors), which we have found to be optimal for promoting proliferation and survival. However, this medium is less suitable for many functional assays, as it keeps endothelial cells in an activated state and limits further activation by external stimuli. We have observed similar limitations with EBM-2, another complex and widely used endothelial medium. Consequently, for most experiments, we use a different formulation: “HIMV” (an AIM-V–based medium supplemented with 1% fetal bovine serum and growth factors; referred to as Comp-AIM-V in our previous publications^8–10^), which supports the formation of a stable, confluent, and well-functioning endothelial monolayer. Since the precise composition of AIM-V is protected by a patent, we use an alternative serum- and growth factor–restricted version of MCDB—referred to as 'MCDB-Steady’ or 'MCDB-S'—in experiments where defined media constituents are required.

In vitro experimental setups involving primary cells are subject to an additional important variable that may partly account for the observed variance of the results: the genetic background of the donor. A striking example from our laboratory is a study by Kiszel et al., which demonstrated that LPS or IL-1β induced IL-6 production by HUVECs can differ by orders of magnitude depending on the donor^11^. Moreover, Lorenz et al. reported sex-specific differences in HUVECs derived from female–male twin pairs^12^, providing compelling evidence of genetic background effects.

**Fig. 1:**
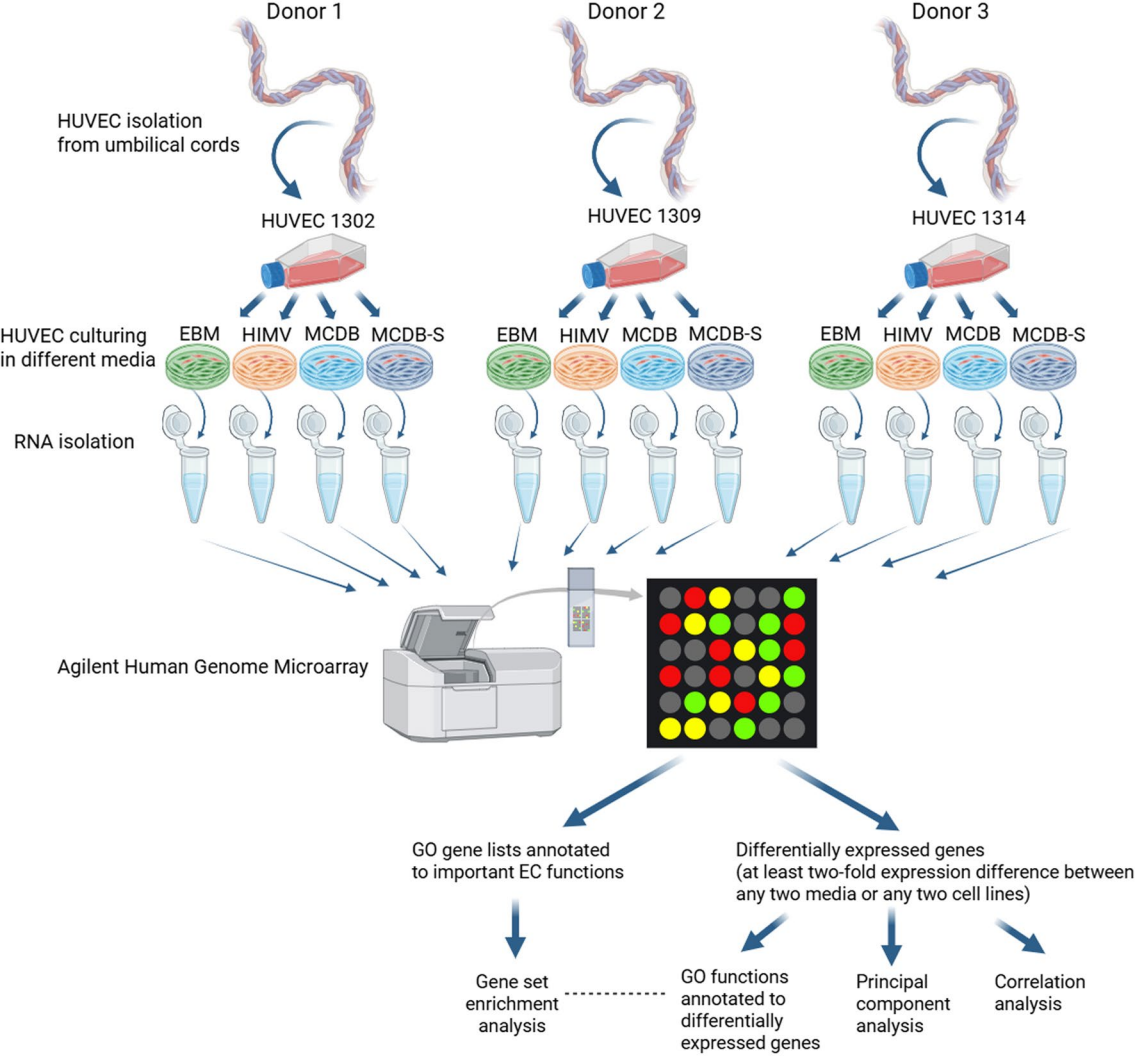
Experimental setup of the transcriptomic analysis. Three well-characterized human umbilical vein endothelial cell (HUVEC) lines from unique donors (HUVEC 1302, 1309 and 1314) were cultured in four different media (EBM, HIMV, MCDB and MCDB-S, the exact compositions of which are presented in Table 1). MCDB and EBM-2 (both rich in serum and growth factors; commercially available) are optimized for cell proliferation and maintaining endothelial cells in an activated state. In contrast, HIMV (AIM-V–based, low serum, exact composition undisclosed) and MCDB-S (with defined, limited serum and growth factors) promote a quiescent monolayer better suited for functional assays. HUVECs were first cultured to confluency in MCDB medium, then exposed to a 50:50 mixture of MCDB and one of four media (EBM, HIMV, MCDB, MCDB-S) for 24 h, followed by 100% of the respective medium for an additional 24 h before mRNA isolation. We performed genome-wide mRNA expression profiling of the 12 samples using the Agilent microarray platform and analyzed the data using a comprehensive set of bioinformatic approaches. Created in https://BioRender.com.

Given these considerations, understanding the relative contributions of culture media composition (Table 1) and donor genetic heterogeneity to experimental variability is essential for optimizing experimental design and improving the reproducibility of in vitro studies. For this purpose, we employed a whole-transcriptome microarray approach to analyze three individual HUVEC lines cultured in the four distinct media described above and subjected the resulting mRNA expression data to a diverse array of bioinformatic analyses (Fig. 1).

**Table 1:** Components of the four cell culture media used in the experiments.

Basal media		EBM	HIMV	MCDB	MCDB-S
		EBM-2 basal medium (Lonza)	AIM-V (life technologies)	MCDB-131 (life technologies)	MCDB-131 (life technologies)
Originally suggested for		Human microvascular endothelial cells	Monocytes, Dendritic Cells, T Cells, Hybridomas, PBMCs, Fibroblasts, Macrophages, Myeloma Cells, Lymphocytes	Human microvascular endothelial cells	Human microvascular endothelial cells
Additives	Heat-inactivated fetal calf serum (FCS, PAN Biotech)	5%	1%	5%	1%
	Antibiotics	30 μg/ml gentamicin and 15 ng/ml amphotericin	(50 μg/ml streptomycin and 10 μg/ml gentamicin)	100 U/ml penicillin and 100 μg/ml streptomycin	100 U/ml penicillin and 100 μg/ml streptomycin
	Heparin	–	7.5 U/ml	7.5 U/ml	7.5 U/ml
	Chemically Defined Lipid Concentrate (Life Technologies)	–	–	1%	1%
	Glutamax (Life Technologies)	–	–	1%	1%
	Ascorbic acid	75 μg/ml	–	5 μg/ml	5 μg/ml
	Hydrocortisone	0.2 μg/ml	–	250 nM	250 nM
	HEPES	–	–	10 mM	10 mM
	Insulin Transferrin Selenium (Life Technologies)	–	–	0,3%	–
	Human recombinant epidermal growth factor (hrEGF, R&D Systems)	10 ng/ml	2 ng/ml	2 ng/ml	–
	Human recombinant basic fibroblast growth factor (hrBFGF)	4 ng/ml	1 ng/ml	1 ng/ml	–
	VEGF	2 ng/ml	–	–	–
	R3-IGF-1	5 ng/ml	–	–	–

The table lists only the concentrations of additives used to supplement the basal media, except for antibiotics, where the manufacturer’s additives are presented in parentheses.

Our comprehensive transcriptomic analysis employing primary endothelial cells underscores the crucial importance of cell culture medium over donor genetic background—a finding likely applicable to other primary cell types cultured in vitro.

## Results

The three HUVEC lines were confirmed to exhibit endothelial identity and function, with consistent proliferation, marker expression, and morphology, as well as appropriate activation of signaling pathways and increased permeability in response to pro-inflammatory stimuli (For further details, see Supplementary Material 1, Supplementary Fig. S1).

### Genome-wide cluster analysis (GWCA)

**Fig. 2:**
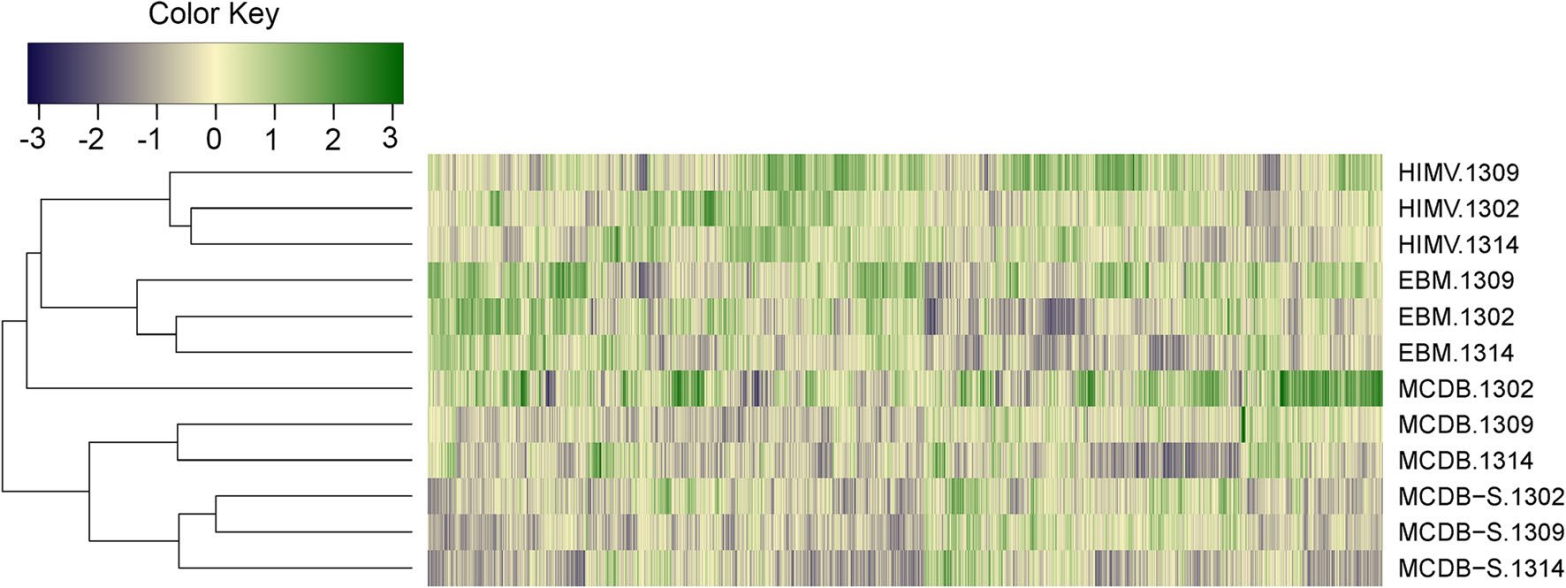
Genome-wide cluster analysis. The z scores of the logarithmically transformed fluorescence intensity values of the whole transcriptome of HUVECs (n = 13,823) were plotted on a color scale. The dendrogram shows the hierarchical clustering of the 12 samples, which was performed using the heatmap.2 function in R. The clustering was based on the Euclidean distance between the samples, with the complete linkage method applied.

We performed GWCA including the whole transcriptome of HUVECs (n = 13,823), to see whether the genetic background or the different culture conditions alter the transcriptomics profile of the cells more (Fig. 2). Except for the MCDB 1302 sample, all samples were clustered by media rather than the cell lines (i.e. donor genetic background). MCDB and its restrictive version, MCDB-S, were the most similar to each other, whereas EBM and HIMV media appeared to be distinct from them.

### Identification of significantly altered genes between media and cell lines

**Fig. 3:**
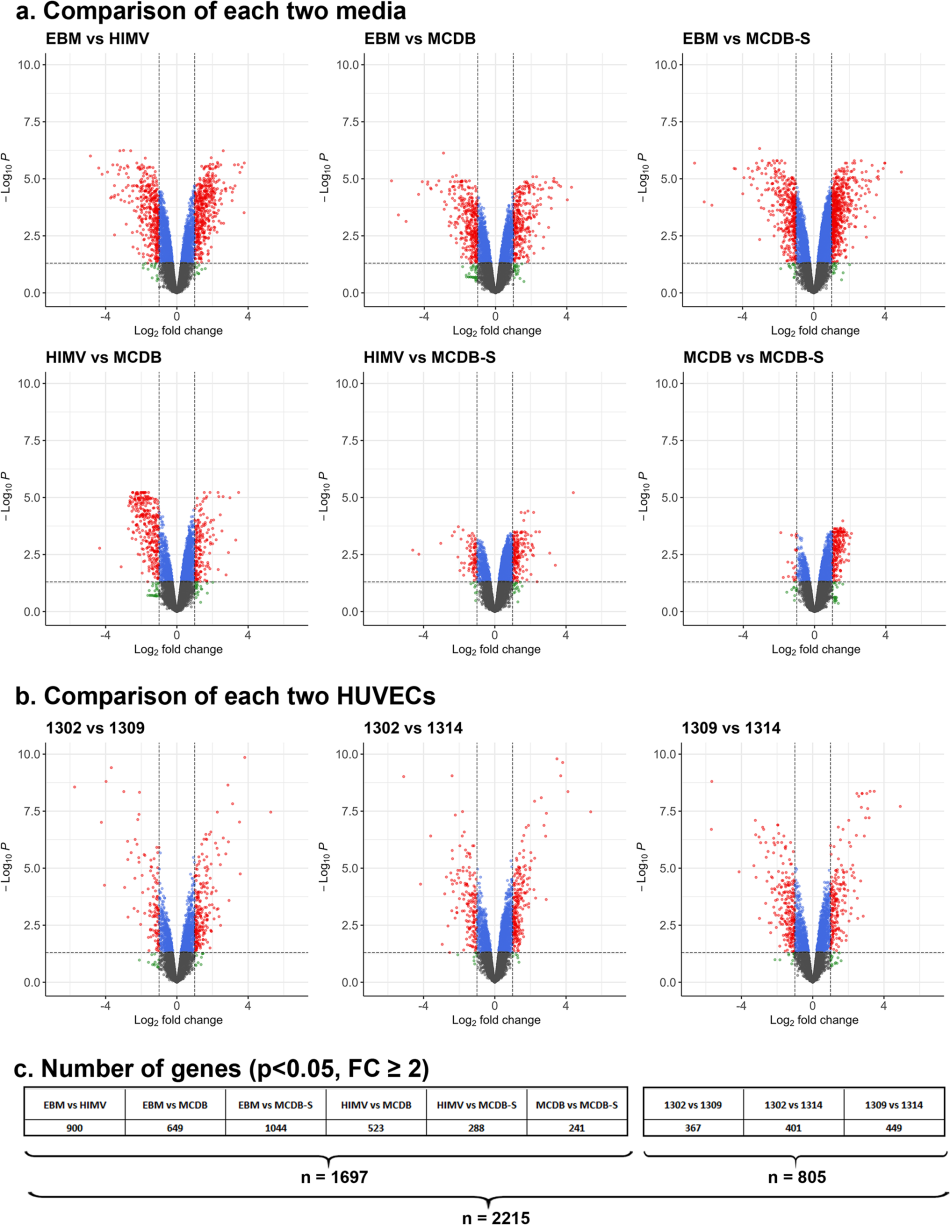
Visualization of gene expression differences between pairs of media (panel a) and between pairs of HUVECs (panel b) on Volcano plots. For media comparisons, HUVEC lines cultured in the same medium were treated as replicates. Conversely, for HUVEC comparisons, the four different cell culture media served as replicates. The log2 transformed fold change (FC) values and the -log10 of the adjusted p-values (Benjamini–Hochberg method) of the genes were plotted for each comparison. Genes are color coded as follows: green dots: FC ≥ 2 and p-value > 0.05; blue dots: FC < 2 and p-value < 0.05; red dots: FC ≥ 2 and p-value < 0.05; grey dots: FC < 2 and p-value > 0.05. Panel c displays the number of genes that showed a significant change (p < 0.05) of at least two-fold for each comparison.

After clustering the samples based on the whole transcriptome of HUVECs, we aimed to find genes with significantly altered expression either between cell culture media or between cell lines. For media comparisons, the three HUVEC lines cultured in the same medium were considered replicates, whereas for HUVEC comparisons, the four different culture media were treated as replicates. To assess differential gene expression, we compared every pair of media and every pair of cell lines using linear model fitting and visualized the data on Volcano plots (Fig. 3). The log2 transformed fold change (FC) values and the adjusted p-values of the significantly altered genes are presented in Supplementary Material 2. Based on the number of significantly altered genes (Fig. 3c), the effect of media composition on the transcriptome of HUVECs was greater than the effect of genetic heterogeneity. MCDB-S was most similar to MCDB and HIMV, while EBM stood out as the most distinct from all other media. In contrast, the three cell lines diverged to a similar extent. For further analysis, we collected the genes that exhibited a significant change (adjusted p value < 0.05) of at least two-fold between any two media (n = 1697) or between any two cell lines (n = 805). For specific analyses, these two categories were combined, resulting in a total of 2215 genes that were subsequently analyzed. To enable a direct comparison between media and cell line effects—despite the unequal number of comparisons—we calculated the average [± SD] number of genes significantly altered per pairwise comparison of media (607,5 [± 322,9]) or cell lines (405,7 [± 41, 2]).

From each comparison, the top three genes with the highest fold change (FC) values were collected and summarized in Supplementary Table S1. In the comparison of different media, several genes were associated with inflammation and permeability regulation (*CXCR4, CCL2, MMP1, UBD, NPPB*), while others were linked to metabolic reprogramming and cell growth (*IGFBP5, NEK2, DEPDC1, HIST1H1A, NEURL3, CYP1A1*). In the comparison of HUVEC lines, genes associated with immune/inflammatory responses (*FAP*), and cell migration (*LAMC2, COL1A1*) were identified.

Since the 1302 HUVEC line originated from a male newborn, whereas the 1309 and 1314 lines from females, we initially excluded Y chromosome genes and the *XIST* (X-inactive specific transcript) gene from all analyses to standardize the data. However, for quality control purposes, we reincluded these genes and repeated the analysis. Ten Y chromosome genes (D*DX3Y, EIF1AY, IL3RA, KDM5D, NLGN4Y, RPS4Y1, RPS4Y2, TBL1Y, USP9Y, ZFY*) and *XIST* exhibited a significant change (adjusted p-value < 0.05) of at least two-fold between the 1302 and 1309, as well as between the 1302 and 1314 HUVEC lines. In contrast, no significant differences in the Y chromosome genes and the *XIST* gene were observed between the two female (1309 and 1314) cell lines (Supplementary Figure S2).

### Comparing the impact of cell culture media and genetic heterogeneity based on differentially expressed genes

#### Correlation analysis

**Fig. 4:**
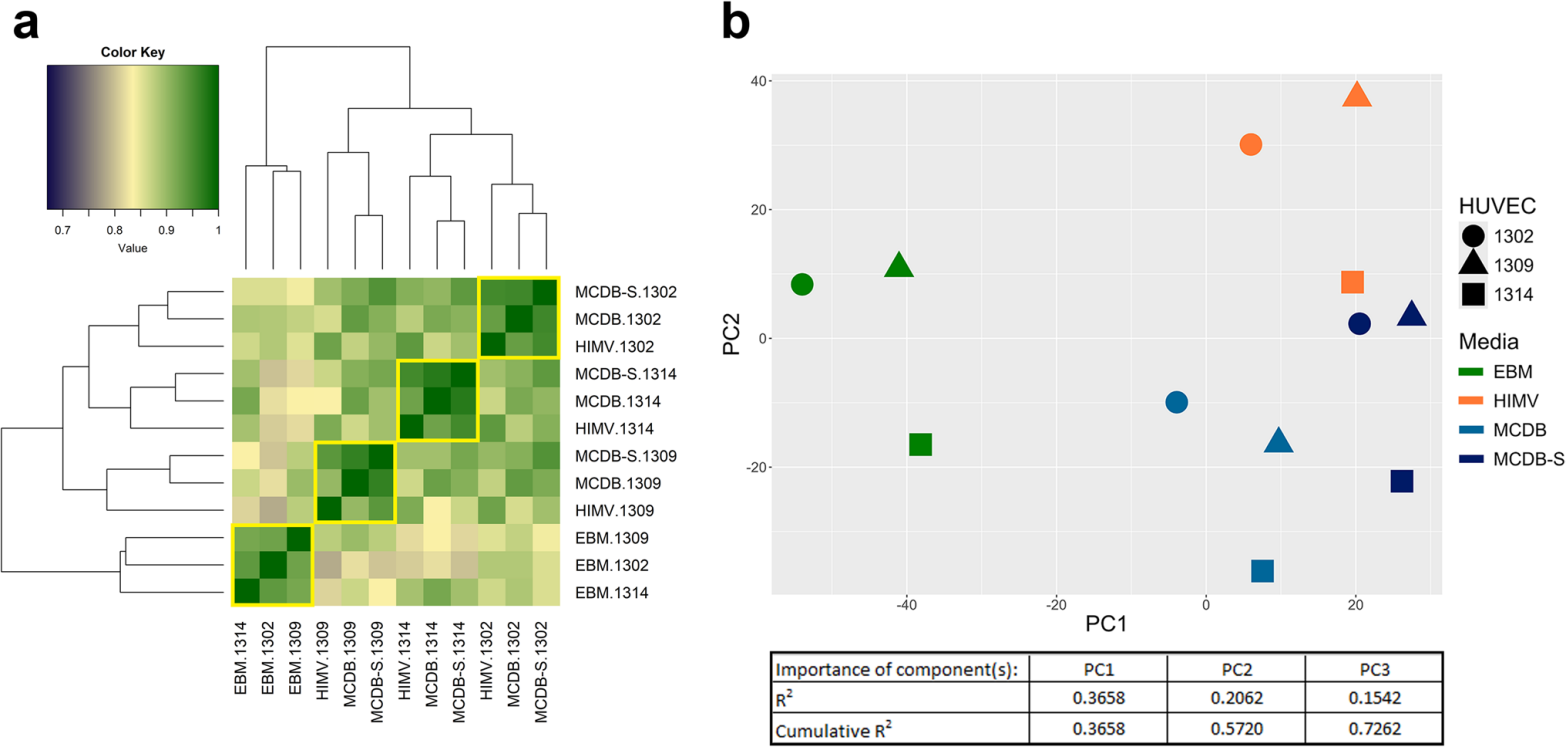
Correlation analysis and principal component analysis (PCA) of significantly altered genes between media or cell lines. Spearman correlation analysis (panel a) was conducted on raw expression values, while PCA (panel b) was performed on z-scores of log-transformed gene expression values of genes that exhibited a significant expression difference (adjusted p-value < 0.05) of at least two-fold between any two media or any two HUVEC lines (n = 2215). A. The Spearman correlation coefficients (r values) are displayed using a color scale and summarized in Supplementary Table S2. The dendrograms show the hierarchical clustering of the 12 samples, which was performed using the heatmap.2 function in R. The clustering was based on the Euclidean distance between the samples, with the complete linkage method applied. B. Three principal components (PCs) were calculated (and their R2 values were plotted), of which the first two were visualized. Different media are represented by distinct colors, while different cell lines are depicted using shapes. (The importance values of the genes in each principal component (PC) are provided in Supplementary Material 3).

After obtaining the lists of significantly altered genes, we aimed to evaluate whether the effect of cell culture media or genetic heterogeneity is greater based on them. When genes significantly altered between media (n = 1697) and those between cell lines (n = 805) were analyzed separately, the samples clustered exclusively by media or by cell line, respectively, as expected (Supplementary Figure S3). When we analyzed the expression correlations of all genes that exhibited a significant change of at least two-fold between any two media or any two cell lines (n = 2215) (Fig. 4A), the samples showed a mixed clustering pattern influenced by both the culture medium and the donor background. EBM medium formed a clearly distinct cluster, whereas the other three media types (HIMV, MCDB, and MCDB-S) could not be separated as clearly, since they tended to group primarily according to cell lines. This indicates that, while culture conditions exert a strong influence, donor-specific genetic heterogeneity also substantially contributes to the clustering pattern. As the choice of a two-fold change cutoff was arbitrary, we repeated the correlation analysis using a 1.5-fold change threshold and observed very similar clustering patterns (Supplementary Figure S4). The Spearman correlation coefficients (r values) are presented in Supplementary Table S2 for each analysis.

#### Principal component analysis

To further evaluate the influence of cell culture media and genetic variability on the cells, we performed principal component analysis (PCA) using all genes that showed a significant change of at least two-fold between any two media or any two cell lines (n = 2215). The three calculated principal components (PCs) explained 72.62% of the cumulative variance. According to PCA, the 12 samples were grouped by media rather than by cell lines (Fig. 4b). PC1 alone could distinguish EBM from the other three media types, whereas in combination with PC2, they effectively discriminated among all four media. The three genes that contributed most significantly to PC1 *were ST6GALNAC3, TAP1,* and DRAM1, whereas the genes with the greatest contribution to PC2 *were IRS2, DDIT3*, *and LAMA3*. The importance values of the genes in each PC are shown in Supplementary Material 3. MCDB and MCDB-S were the closest to each other, consistent with the results from the genome-wide cluster and correlation analyses. HIMV was the second closest to MCDB-S, and EBM was the most distinct from all other media. Similar to the correlation analysis, we performed PCA separately for genes significantly altered between media (n = 1697) and those significantly altered between cell lines (n = 805). In each case, the samples clustered by media or by cell lines, respectively (Supplementary Figure S5).

### Linking transcriptomics profiles to key endothelial cell functions

Next, we sought to identify which endothelial cell (EC) functions could be influenced by altered gene expression. To achieve this, we employed two distinct approaches. The first approach involved using the previously obtained gene lists of significantly altered genes between media (n = 1697) or cell lines (n = 805) and associating them with terms from the Gene Ontology (GO), KEGG and REACTOME databases, using g:Profiler. Additionally, we applied the unbiased method of Gene Set Enrichment Analysis (GSEA), which compares the expression of genes within predefined gene sets to the entire transcriptome across two samples, identifying statistically significant differences in the activity of specific gene sets between the conditions. For GSEA, nine gene sets related to key endothelial cell functions (mitotic cell cycle, epithelial to mesenchymal transition, angiogenesis, regulation of cell adhesion, inflammatory response, programmed cell death, hemostasis, and regulation of vascular permeability) were obtained from the Gene Ontology database. The names of these gene sets were then used as category labels when organizing the terms from the g:Profiler analysis, allowing for a direct comparison between the two approaches.

#### g:profiler analysis

**Fig. 5:**
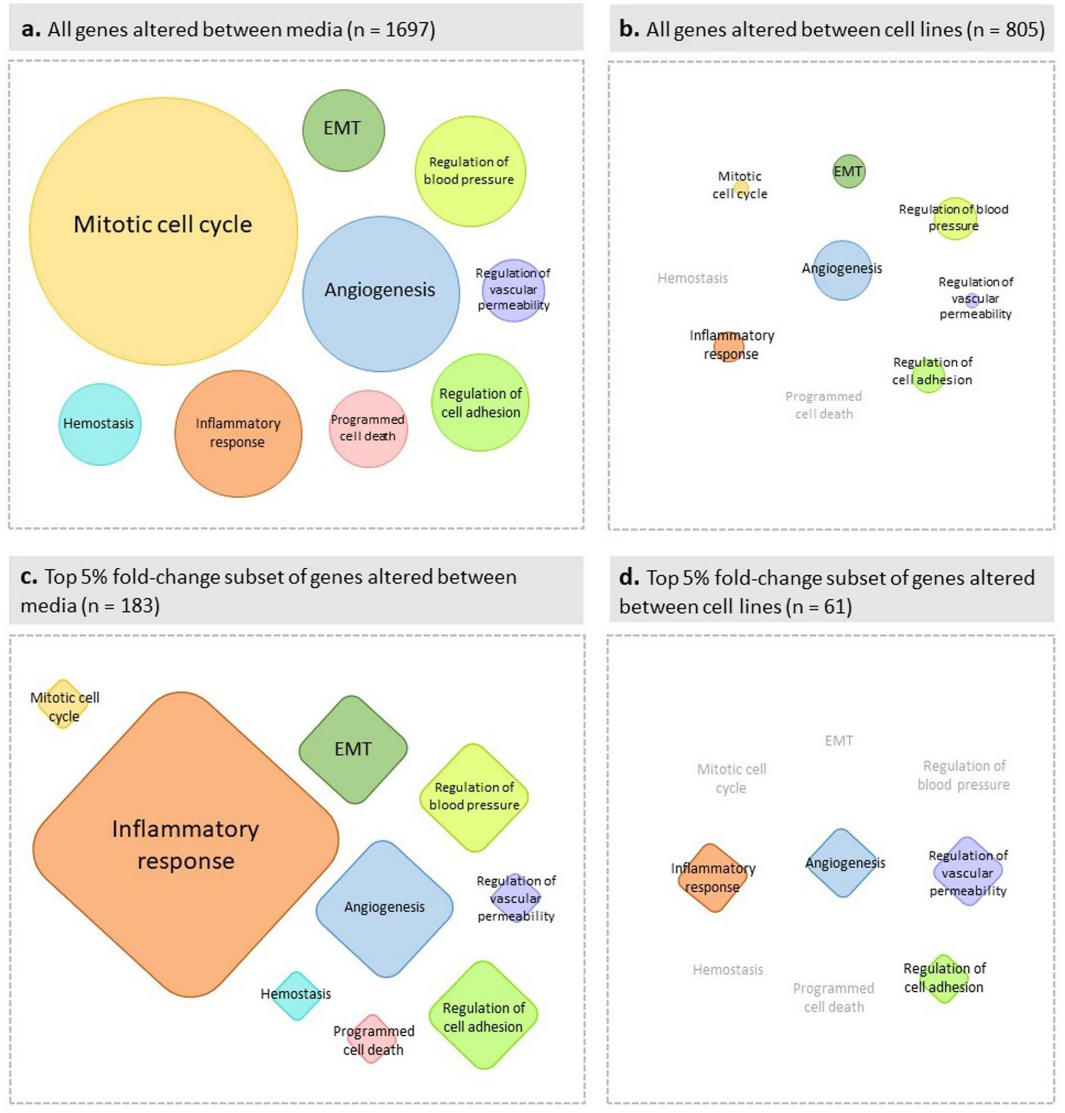
Visualization of EC functions influenced by altered gene expression between cell culture media and cell lines. Sets of all significantly altered genes (adjusted p-value < 0.05) exhibiting at least a twofold change either between any two media (panel a) or between any two cell lines (panel b), and the top 5% fold-change subsets of them (panel c and d) were analyzed by g:Profiler. The resulting lists of enriched GO, KEGG and REACTOME terms for the four analyses (see Supplementary Table 3) were manually categorized into nine key endothelial cell function groups. The areas of the circles and squares are proportional to the number of terms associated with each endothelial cell function category, though the two shapes are scaled differently for better visualization. EMT: epithelial to mesenchymal transition.

To compare whether culture conditions or genetic heterogeneity has a greater influence on selected endothelial functions, we mapped the significantly altered genes between media (n = 1697) or cell lines (n = 805) separately to terms from the Gene Ontology (GO), KEGG, and REACTOME databases. The lists of enriched terms are presented in Supplementary Material 4. For clarity, these enriched terms were manually categorized into the above-mentioned nine key endothelial cell function groups (Fig. 5). Notably, the number of terms enriched for genes influenced by cell culture media (Fig. 5a) was markedly higher than those enriched for genes affected by genetic heterogeneity (Fig. 5b). Cell culture media influenced all nine selected endothelial functions, with the strongest enrichment observed for terms related to the mitotic cell cycle and angiogenesis. In contrast, genetic heterogeneity appeared to affect only seven of the nine functions (angiogenesis, regulation of cell adhesion, inflammatory response, epithelial to mesenchymal transition, regulation of blood pressure, and regulation of vascular permeability), and to a much lesser extent as represented by smaller circles on Fig. 5.

Certain biological processes are shaped by subtle yet coordinated changes in the expression of numerous genes, whereas other cellular functions are governed by pronounced changes in a smaller set of genes. To capture the latter pattern in addition to the former, we mapped the top 5% fold-change subsets of the significantly altered gene sets (media: n = 183; cell lines: n = 61) to GO, KEGG, and REACTOME terms (see Supplementary Material 4), as described previously. Consistent with our earlier findings, the number of terms enriched for genes influenced by cell culture media (Fig. 5c) was markedly higher than those for genes affected by genetic heterogeneity (Fig. 5d) within the top 5% fold-change subsets. However, unlike the initial analysis, where terms associated with the significantly altered genes between media were most strongly associated with the mitotic cell cycle, the top 5% fold-change subset of these genes was predominantly associated with the inflammatory response—particularly chemokines, cytokines, and endothelial cell interactions with various immune cells (e.g., granulocytes, lymphocytes, monocytes).

#### Gene set enrichment analysis (GSEA)

**Fig. 6:**
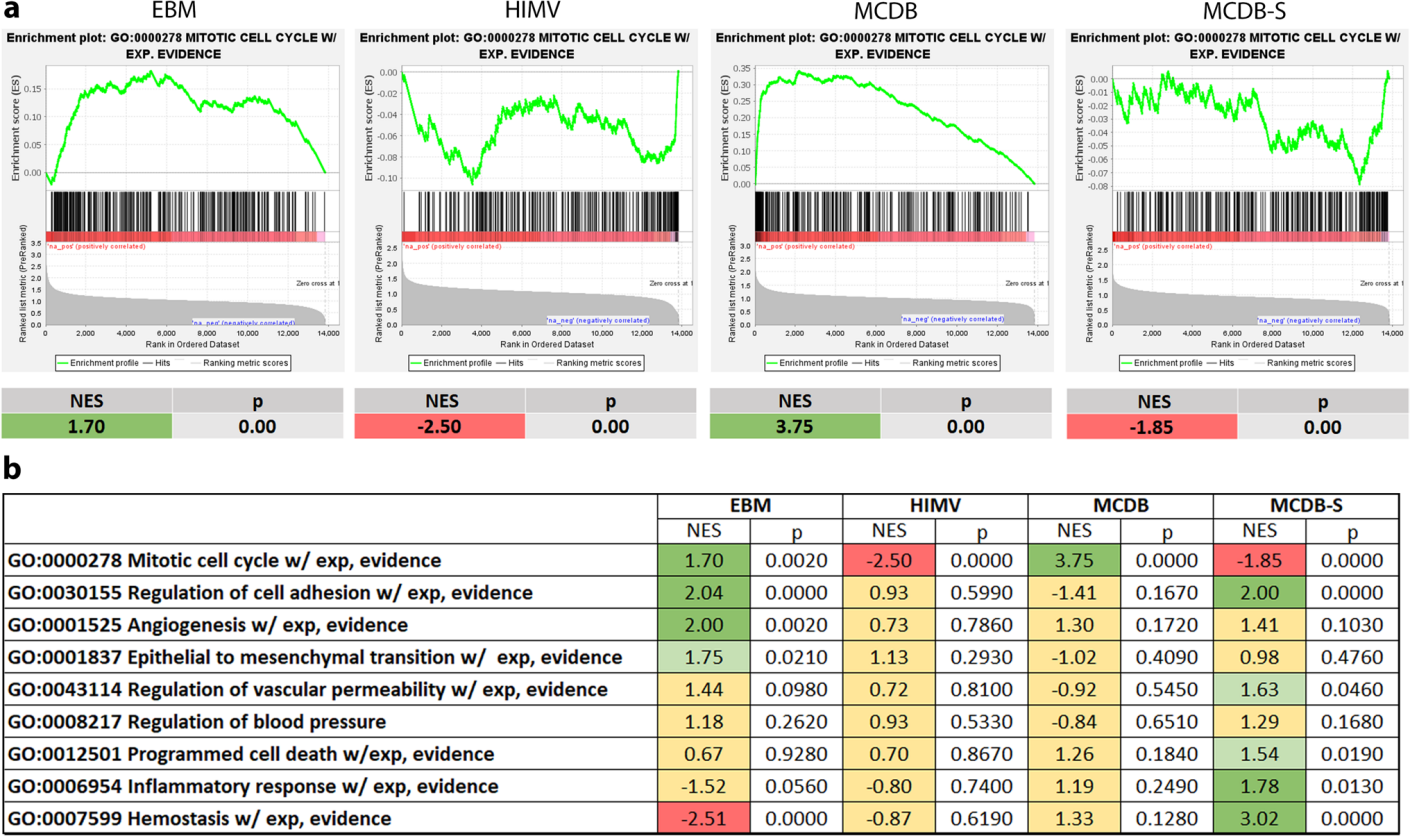
Gene set enrichment analysis (GSEA) of gene sets related to important endothelial cell functions across different cell culture media. Nine gene sets related to key endothelial cell functions were obtained from the Gene Ontology database and filtered to retain only genes supported by experimental evidence. Pre-ranked GSEA was performed using fold change values calculated by dividing the average expression for each medium across all cell lines by the overall average expression across the 12 samples. Panel a shows representative enrichment plots for the mitotic cell cycle gene set, whereas panel b summarizes the GSEA results for all gene sets. Normalized enrichment scores (NESs) and nominal p values are plotted. NESs, colored in green, represent positive enrichment, those in red indicate negative enrichment, whereas yellow signifies no changes. Darker shades highlight changes significant at the p-value threshold (0.0139) set by the Benjamini–Hochberg correction for a 5% false discovery rate, whereas lighter shades denote changes significant only at a p-value threshold of 0.05.

In addition to the g:Profiler analysis, we utilized an unbiased method of GSEA to evaluate the impact of altered gene expression on key endothelial cell functions (Fig. 6). Building on our current findings that media composition has a greater impact on the endothelial cell transcriptome than genetic heterogeneity, our GSEA primarily focused on comparing different cell culture media. (We performed only a limited GSEA as a quality control measure to compare the three HUVEC lines (see Supplementary Material 1, Supplementary Figure S6).) Fold change values were calculated by dividing the average expression for each medium across the three cell lines by the overall average expression across the 12 samples. The mitotic cell cycle gene set was differentially expressed in the four media types. Positive enrichment was observed in EBM (NES = 1.73, p = 0.000) and MCDB (NES = 3.72, p = 0.0000), whereas negative enrichment was seen in HIMV (NES = − 1.82, p = 0.0000) and MCDB-S (NES = − 1.55, p = 0.0000). The expression of four additional gene sets was altered only by EBM and/or MCDB-S, but not by HIMV or MCDB. The regulation of cell adhesion gene set was positively enriched in both EBM (NES = 2.06, p = 0.0000) and MCDB-S (NES = 1.98, p = 0.0000). In contrast, the hemostasis gene set showed positive enrichment in MCDB-S (NES = 3.08, p = 0.000) but negative enrichment in EBM (NES = − 2.55, p = 0.0000). The inflammatory response gene set was positively enriched in MCDB-S (NES = 1.78, p = 0.0130), whereas the angiogenesis gene set was positively enriched in EBM (NES = 2.00, p = 0.0020). The remaining four gene sets exhibited changes at a p-value threshold of 0.05 but did not meet the more stringent threshold (0.0139) set by the Benjamini–Hochberg correction for a 5% false discovery rate. Nonetheless, these alterations may still reflect biologically meaningful changes, particularly when considered alongside the results from the previous g:Profiler analysis.

## Discussion

In our study, we used HUVECs—one of the most commonly employed primary in vitro cell models—to investigate whether the composition of cell culture media or the genetic background, two crucial variables in in vitro experiments, exerts a greater influence on the endothelial cell transcriptome. To ensure the HUVEC lines fully met established endothelial cell criteria, we conducted extensive phenotypic and functional characterization prior to transcriptomic analysis. To generate valid and reproducible results, we applied multiple complementary transcriptomic data analysis approaches. Our results consistently pointed to the superior importance of cell culture media composition compared to donor genetic variability.

Certain aspects of this issue have been addressed previously by other groups: some studies demonstrated that different culture media profoundly affect endothelial cell function^13^, while others, investigating mesenchymal stem cells, MDA-MB-231 breast cancer cells, and murine eosinophils, concluded that culture conditions exert a greater influence on the transcriptomic profile than genetic background^14–16^. In line with these observations, we recently demonstrated that the microRNA signature secreted by endothelial cells is more strongly shaped by culture conditions than by donor-specific genetic variation^17^. In many primary cell types, passage number is also a major source of variability and should be considered; however, this factor is less relevant for HUVECs, which are generally used only at low passages (up to 3–4). Nonetheless, to the best of our knowledge, no prior study has directly compared the relative impact of cell culture media composition and genetic background on the transcriptomic profile specifically in endothelial cells.

Genome-wide cluster analysis revealed that, based on the whole transcriptome, our samples clustered by cell culture media rather than donor genetic background. However, the relative sensitivity of GWCA to outlier data and its inability to capture changes in the expression of individual genes necessitated additional analyses.

The analysis and visualization of data on Volcano plots allowed us to identify differentially expressed genes between pairs of media and between pairs of HUVEC lines. The number of differentially expressed genes was substantially higher between the two most divergent media (1044 genes between EBM and MCDB-S) than between the two most different cell lines (449 genes between HUVEC 1309 and HUVEC 1314), further supporting the importance of media composition. By ranking genes based on their fold change values, we identified individual genes representing the greatest difference between samples. However, it is important to note that the 2 × fold change cutoff was defined arbitrarily, as the biological relevance of expression changes varies from gene to gene.

Gene lists from the Volcano plots were analyzed using two complementary methods (correlation analysis and PCA), both yielding similar results and showing that samples grouped primarily by culture media, with EBM-2 being the most distinct. Correlation analysis quantifies diversity between samples by comparing statistically significant differentially expressed genes, while PCA, a dimensionality reduction technique, identifies individual genes contributing most to the observed variation.

To interpret gene expression differences at the functional level, we performed g:Profiler analysis, which involved manually grouping terms into nine predefined endothelial cell function categories. While some biological processes are governed by subtle changes across many genes, others depend on pronounced changes in a few key genes. These patterns were reflected in two complementary approaches: analyzing all differentially expressed genes revealed that the mitotic cell cycle was most influenced by cell culture media, consistent with our laboratory observations of media-dependent proliferation. In contrast, the top 5% of differentially expressed genes predominantly impacted the inflammatory response rather than the mitotic cell cycle. Consistent with the results of our previously described analyses, far fewer terms were associated with differences between cell lines, and no dominant function emerged.

To address the limitations of the arbitrary twofold expression cutoff used in previous analyses, we applied GSEA, which complemented the g:Profiler results. We predefined 9 key biological processes relevant to endothelial cells and searched for corresponding gene lists in the GO database. GSEA showed that the mitotic cell cycle gene set was differentially expressed in all four media types with a positive enrichment in EBM and MCDB and negative enrichment in HIMV and MCDB-S. This aligns with our laboratory experience: EBM and MCDB support proliferation and are thus best suited for endothelial cell growth, while HIMV and MCDB-S are preferred for maintaining stable monolayers and are therefore more appropriate for functional assays such as Ca^2^⁺ mobilization and permeability tests^8–10^. GSEA further demonstrated that, beyond proliferation, media composition influences other essential endothelial functions, including adhesion, angiogenesis, inflammation, and hemostasis.

While functional analysis provides valuable insights, it is important to acknowledge the limitations of gene set databases such as GO. Even when filtering for human protein-coding genes with experimentally validated roles in predefined biological processes, the gene sets remain incomplete. For example, the ‘regulation of endothelial permeability’ set lacks key regulators such as PECAM1^18^, RhoA, and Rac1^19^, while the ‘inflammatory response’ set is missing several critical mediators, including E-selectin^20^, ICAM-1^21^, and IL-8^22^. Conversely, although it’s inherently more difficult to disprove functional relevance, many gene sets contain genes whose connection to the specific biological function is difficult to explain. Additionally, GO does not indicate the direction of a gene’s influence on a process. Despite these shortcomings, GO and other publicly available databases currently offer the best available tools for objective functional interpretation of gene expression data, though their limitations must be kept in mind.

We identified more than 800 genes that were differentially expressed across the three HUVEC lines, and g:Profiler analysis linked these genes to specific endothelial cell functions. Interestingly, in correlation analysis, donor genetic background was similarly influential as media composition in shaping the clustering pattern. Together, these findings demonstrate that genetic background significantly impacts the transcriptomic profile of primary endothelial cells. Nevertheless, our comprehensive analysis revealed that the composition of the culture medium has an even greater impact on gene expression than donor-specific genetic differences. Although the inclusion of biological replicates from different donors is a widely accepted standard in experiments involving primary cells, the critical role of culture medium selection and its consistent application often receive less attention —despite its substantial influence on experimental outcomes.

In conclusion, besides using primary cells from multiple donors, we emphasize the importance of the conscious choice of cell culture medium.

## Methods

### Cell isolation and culturing

Human umbilical vein endothelial cells (HUVECs) were prepared from fresh umbilical cords of normally delivered healthy neonates by collagenase digestion as described by Oroszlán et al^23^. Cells were cultured in vitro in gelatin-precoated flasks (Corning, Costar) in MCDB medium (Table 1), until reaching full confluency. Then, for the transcriptomics analysis, HUVECs were cultured in four different cell culture media (EBM, HIMV, MCDB, MCDB-S), the compositions of which are summarized in Table 1. Experiments were performed on three independent primary HUVEC cultures from different individuals at passage 3 (namely HUVEC 1302/3, HUVEC 1309/3 and HUVEC 1314/3).

### Characterization of the isolated HUVEC lines

The three isolated HUVEC lines underwent extensive characterization to confirm their endothelial identity. The proliferation rates were comparable among the three HUVEC lines, with a population doubling time of approximately 24 h. In addition to standard endothelial cell characterization—confirming the characteristic ‘cobblestone’ morphology and the expression of key markers (VE-cadherin, PECAM-1, CD34, and Von Willebrand Factor)—we conducted functional tests to ensure the cells exhibited proper endothelial function (Supplementary Fig. S1). Upon stimulation with various pro-inflammatory agents (LPS, IL-1β, thrombin, histamine, and bradykinin), activation of the corresponding inflammatory signaling pathways (NFκB, CREB, and Ca^2^⁺ mobilization) was observed in each cell line. Furthermore, all three HUVECs exhibited a significant increase in permeability in response to thrombin stimulation. A detailed description of the functional tests (signaling pathway experiments and permeability test) is provided in the methods section of Supplementary Material 1.

### Total mRNA isolation and whole genome microarray

Confluent layers of three individual HUVEC lines (1302, 1309, 1314) were cultured in four cell culture media (EBM, HIMV, MCDB, MCDB-S) in 24-well plates for 1 days. HUVECs were washed with PBS, lysed and stored in TRI® reagent (Merck-Sigma), followed by total RNA extraction from the 12 samples, using the SPLIT RNA Extraction Kit (Lexogen). mRNA expression was measured on the Agilent Microarray platform, with all RNA samples having RIN values above 9. Samples were processed according to the Agilent Two-Color Microarray-Based Gene Expression Analysis Low Input Quick Amp Labeling Kit protocol, using the Agilent Spike-In Kit as per the manufacturer’s instructions. The detailed protocol is available at: https://www.agilent.com/cs/library/usermanuals/public/G4140-90050_GeneExpression_TwoColor_6.9.pdf. Technical replicates were not included for each donor–culture medium combination, as the technical performance of the Agilent microarray platform had been previously evaluated in an independent transcriptomic experiment conducted by our group (unpublished). In that validation experiment, four technical replicates per condition yielded a median coefficient of variation of 18% after normalization, indicating good technical reproducibility, consistent with Agilent’s recommendations for evaluating microarray performance (https://www.manuallib.com/download/pdf11/AGILENT-EVALUATING-THE-REPRODUCIBILITY-OF-MICROARRAY-TECHNOLOGY-MANUAL.PDF). Equal amounts of Cyanine-3 (Cy3)-labeled or Cyanine-5 (Cy5)-labeled cRNA from samples were co-hybridized onto arrayed oligonucleotides on the same Agilent SurePrint G3 Human GE v3 8 × 60 K (GPL21185) slide at 65 °C for 17 h using the Agilent Gene Expression Hybridization Kit in SureHyb Hybridization Chambers (G2545A). Post-hybridization, the microarrays were washed as per the manufacturer’s protocol and scanned using an Agilent Microarray Scanner (G2505C) with 2 μm resolution and 20-bit color depth. The scanned images were processed and analyzed using Agilent GeneSpring 14.5-GX software.

### Pre-processing transcriptomics data

The measured Cy5 and Cy3 signals of a total of 20,672 protein encoding genes passed the built-in QC analysis of the software executed according to the manufacturer’s protocol for two-color experiments. All data are available at the ArrayExpress database at the European Nucleotide Archive (ENA) under the series accession number [E-MTAB-15672]. Fluorescence intensity values for 14 housekeeping genes (HKGs: RPLP0, PGK1, PPIA, SDHA, GAPDH, RPL13A, UBE2D2, HPRT1, YWHAZ, TFRC, ACTB, B2M, TBP, and HMBS) were collected across the 12 samples. Data normalization was performed based on the average of the first 12 HKGs listed, as these exhibited the lowest standard deviation across the samples. Fluorescence intensity values across the 12 samples were subsequently collected for another 12 genes—hereafter referred to as ‘negative genes’—selected based on their well-established use as lineage-restricted or cell type–specific markers for non-endothelial cell populations, prior qPCR analyses, and cross-referencing with publicly available transcriptomic resources (Human Protein Atlas and GTEx), and therefore not expected to be expressed in HUVECs (KLRD1, CD79B, FCGR1A, PTPRC, ELANE, CD3E, MS4A1, IL2, TNNT2, RHO, ACE2, and SIGLEC5)^24–26^. The detection limit was set at 95th percentile (36.71) of the fluorescence intensity values of these 'negative genes’ across the 12 samples. Genes were filtered out if they did not meet any of the following criteria: (1) expression above the detection limit in at least 4 samples; (2) expression above twice the detection limit in at least 2 samples; or (3) expression above three times the detection limit in at least 1 sample. The 1302 HUVEC line originated from a male newborn, whereas the 1309 and 1314 lines from females. To standardize the data, we excluded Y chromosome genes and the *XIST* (X-inactive specific transcript) gene from further analysis. After pre-processing, 13,823 genes remained and were included in the subsequent analyses. This set of genes was later referred to as the whole transcriptome of HUVECs.

### Defining gene sets related to key endothelial cell functions

To link the transcriptomic profiles of HUVECs to potential functional outcomes, we arbitrarily selected nine key endothelial cell functions based on predefined Gene Ontology (GO) terms: mitotic cell cycle (GO:0,000,278), angiogenesis (GO:0,001,525), regulation of cell adhesion (GO:0,030,155), inflammatory response (GO:0,006,954), epithelial-to-mesenchymal transition (GO:0,001,837), programmed cell death (GO:0,012,501), hemostasis (GO:0,007,599), regulation of vascular permeability (GO:0,043,114) and regulation of blood pressure (GO:0,008,217). The gene sets corresponding to each function were created by filtering the genes annotated to each GO term to only include human, protein encoding genes with direct experimental evidence supporting their relevance to the specific function.

### Statistical analysis

Statistical analyses were carried out using the R v4.4.0 programming language in RStudio v2024.04.1. Hierarchical clustering was based on the Euclidean distance between the samples, with the complete linkage method applied.

Volcano plots were generated using the EnhancedVolcano 1.22.0 R package, following liner model fitting using the limma 3.60.6 R package. For media comparisons, the three HUVEC lines cultured in the same medium were considered replicates, whereas for HUVEC comparisons, the four different culture media were treated as replicates. The p values were adjusted for multiple testing using the Benjamini–Hochberg method.

Principal component analysis (PCA) was performed on z-scores of log-transformed gene expression values, while Spearman correlation analysis was conducted on raw expression values of genes that exhibited a significant change (adjusted p-value < 0.05) of at least two-fold between any two media or any two HUVEC lines (n = 2215). PCA utilized the pcaMethods 1.96.0 R package, and correlations were calculated using the Hmisc 5.2–0 R package. The number of principal components (PCs) was set to three for each comparison, ensuring that the final PC accounted for at least 5% of the variance. Data visualization was done using the gplots 3.2.0 and ggplot2 3.5.1 R packages.

For the comparison of different cell culture media, preranked gene set enrichment analysis (GSEA) was performed using GSEA version 4.3.2 from the Broad Institute (MIT)^27^, analyzing the above described nine gene sets, obtained from the Gene Ontology database. Fold change values were calculated by dividing the average expression for each medium across the three cell lines by the overall average expression across the 12 samples. Normalized enrichment scores (NESs) and nominal *p* values were calculated, and p-value thresholds were set after applying a 5% false discovery rate correction using the Benjamini–Hochberg method.

## Supplementary Information

Below is the link to the electronic supplementary material.

Supplementary Material 1.

Supplementary Material 2.

Supplementary Material 3.

Supplementary Material 4.

## Data Availability

The raw microarray data generated and analysed during the current study are available in the ArrayExpress repository under accession number E-MTAB-15672 (https://www.ebi.ac.uk/biostudies/arrayexpress/studies/E-MTAB-15672#) and are publicly accessible as of 2 March 2026. All original code has been deposited at Zenodo (10.5281/zenodo.17226279) and is publicly available as of the date of publication. Any additional information required to reanalyze the data reported in this paper is available from the lead contact, Flóra Demeter (demeter.flora@semmelweis.hu) upon request.
