# Supplementary material for: Nature versus nurture: genetic background and media composition shape endothelial cell transcriptomes in vitro
Source: Sci Rep. 2026 Mar 16;16:13621. doi: 10.1038/s41598-026-43732-0 (PMC13121701; doi:10.1038/s41598-026-43732-0)
Supplement: Supplementary file 1 — Supplementary Material 1. [file 41598_2026_43732_MOESM1_ESM.pdf]

Nature versus nurture: Genetic background and media composition shape endothelial cell transcriptomes in vitro

Author affiliations and footnotes

Flóra Demeter[1,4,*], Márta Lídia Debreczeni[1,2,4], Zsuzsanna Németh[1], Erika Kajdácsi[1,2], Zoltán Doleschall[1,3], László Cervenak[1]

1 Department of Internal Medicine and Hematology, Semmelweis University, Budapest, Hungary

2 Research Group for Immunology and Hematology, Semmelweis University-HUN-REN-SU (Office for Supported Research Groups), Budapest, Hungary

3 Department of Pathogenetics, National Institute of Oncology, Budapest, Hungary

4 These authors contributed equally

SUPPLEMENTARY MATERIAL

SUPPLEMENTARY METHODS

Characterization of the isolated HUVEC lines

Intracellular Ca^{2+} mobilization assay

Intracellular Ca^{2+} mobilization was assessed as previously described by our group [1]. Briefly, HUVECs were cultured in HIMV media to confluence in 96-well plates for 48h. The cells were then loaded with 2 µM of Fluo-4-AM for 20 min, followed by a 20-minute incubation in HBSS (Life Technologies). An Olympus IX-81 microscope mounted by an Olympus XM-10 camera was utilized for fluorescence imaging. To determine baseline fluorescence, three images were captured before the addition of Thrombin (3 U/mL), Histamine (5 µM) or Bradykinin (2 µM). Then sequential images were taken every 5 seconds for 2 min. Fluorescence intensity changes compared to the baseline fluorescence were calculated by analyzing at least 20 cells per image using CellP software (Olympus).

Visualization of inflammatory signaling

For pCREB and NFκB experiments, confluent monolayers of HUVECs cultured in HIMV media for 48 hours. Cells were treated with 1 µg/mL of LPS, 1 ng/mL of IL-1β or left untreated for 30 min. After treatment, cells were fixed in methanol-acetone (1:1) and stained with rabbit-anti-human phospho-CREB (pCREB, 1:200, Cell Signaling Technology) or rabbit-anti-human NFκB (p65 subunit, 1:250, Santa Cruz) followed by Alexa Fluor568-conjugated goat anti-rabbit (1:500, SouthernBiotech) IgG, Alexa Fluor568-conjugated wheat germ agglutinin (WGA, 1:200, Life Technologies) and Fluorescence images were captured using an Olympus IX-81 microscope with an Olympus XM-10 camera. Image analysis was performed using CellP software (Olympus), and nuclear mean red fluorescence (for pCREB) or the difference between cytoplasmic and nuclear mean red fluorescence (for NFκB) was calculated.

Measurement of paracellular permeability

Paracellular permeability assays were conducted using the XPerT method [2], with slight modifications by Debreczeni et al. [3]. HUVECs were cultured to confluence in 96-well plates pre-coated with 250 µg/ml biotinylated gelatin for 2 days in HIMV media. After a 20-minute treatment with thrombin (3 U/mL), Streptavidin-Alexa488 (2 µg/ml) was added to each well for 2 minutes, followed by fixation with 1% paraformaldehyde-HBSS. The plates were then analyzed using a fluorescent plate reader (TECAN Infinite M1000 PRO) to assess the ratio of stained areas.

Gene set enrichment analysis

For comparing cell lines, pre-ranked gene set enrichment analysis (GSEA) was performed using GSEA version 4.3.2 from the Broad Institute (MIT) (PMID: 16199517), analyzing only the regulation of vascular permeability gene set. Fold change values were determined by dividing the expression of each cell line in HIMV medium by the average expression of the three cell lines in HIMV medium, allowing for direct comparison with the functional permeability tests that were carried out in HIMV. Normalized enrichment scores (NESs) and nominal p values were calculated, and p-value thresholds were set after applying a 5% false discovery rate correction using the Benjamini–Hochberg method.

SUPPLEMENTARY RESULTS

Gene set enrichment analysis

For the comparison of cell lines, only the 'regulation of vascular permeability' gene set was analyzed. This focus was driven by the functional permeability measurements conducted for each cell line, allowing for a direct comparison with the GSEA results. The permeability measurements were performed in HIMV medium, so fold change values for GSEA were calculated by dividing the expression of each cell line in HIMV medium by the average expression of the three cell lines in the same medium. The regulation of vascular permeability gene set showed no significant enrichment in any of the three HUVEC lines (Figure S6), consistent with our previous finding that permeability changes were comparable across the three lines.

SUPPLEMENTARY FIGURES

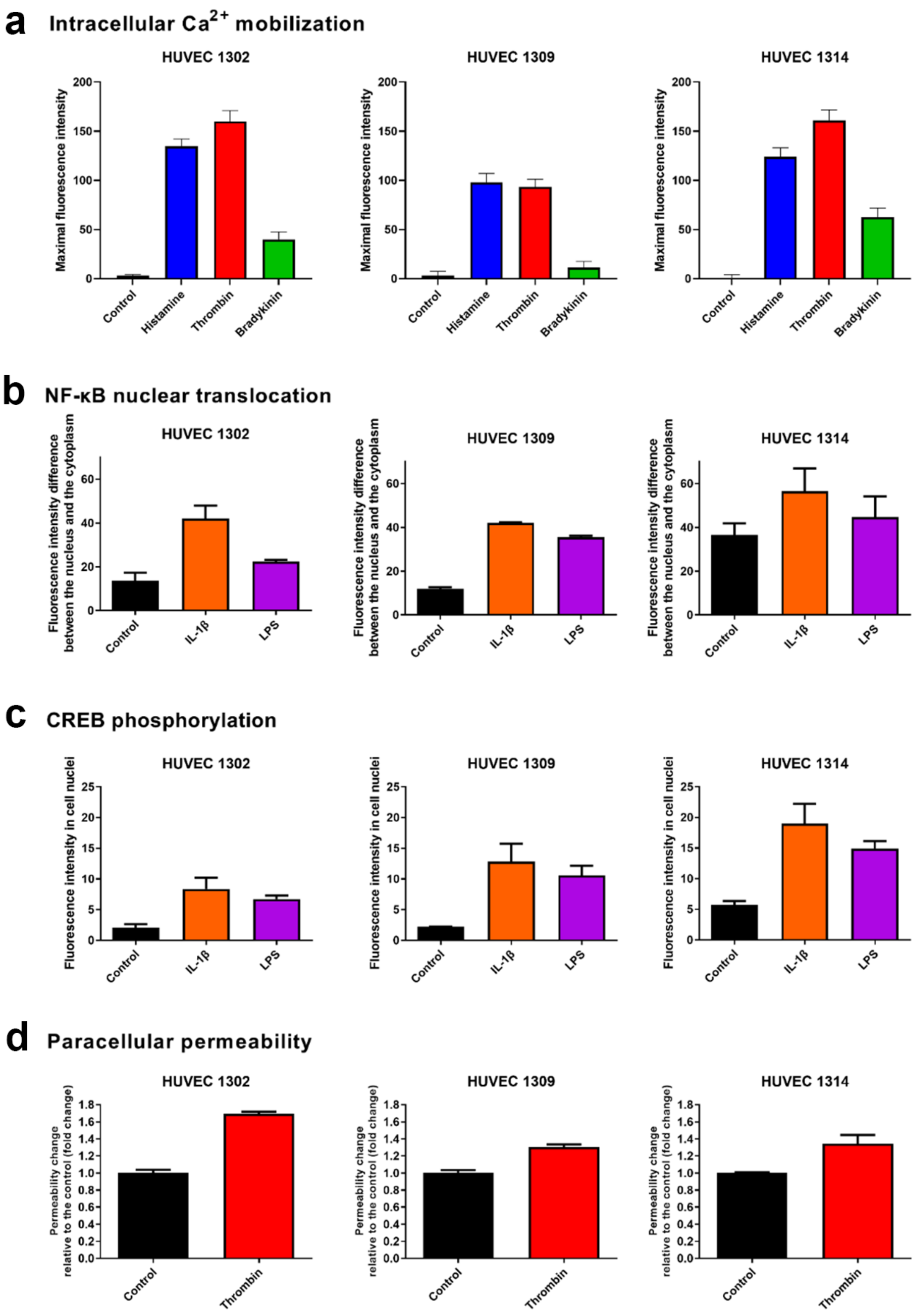

Figure S1. Functional tests: To confirm the normal functionality of our HUVEC lines, we measured intracellular Ca^{2+} mobilization (a), NFκB nuclear translocation (b), CREB phosphorylation (c) and changes in paracellular permeability (d) upon stimulation of HUVECs with various pro-inflammatory agents (3 U/mL of thrombin, 5 µM of histamine, 2 µM of bradykinin, 1 µg/mL of LPS, and 1 ng/mL of IL-1β).

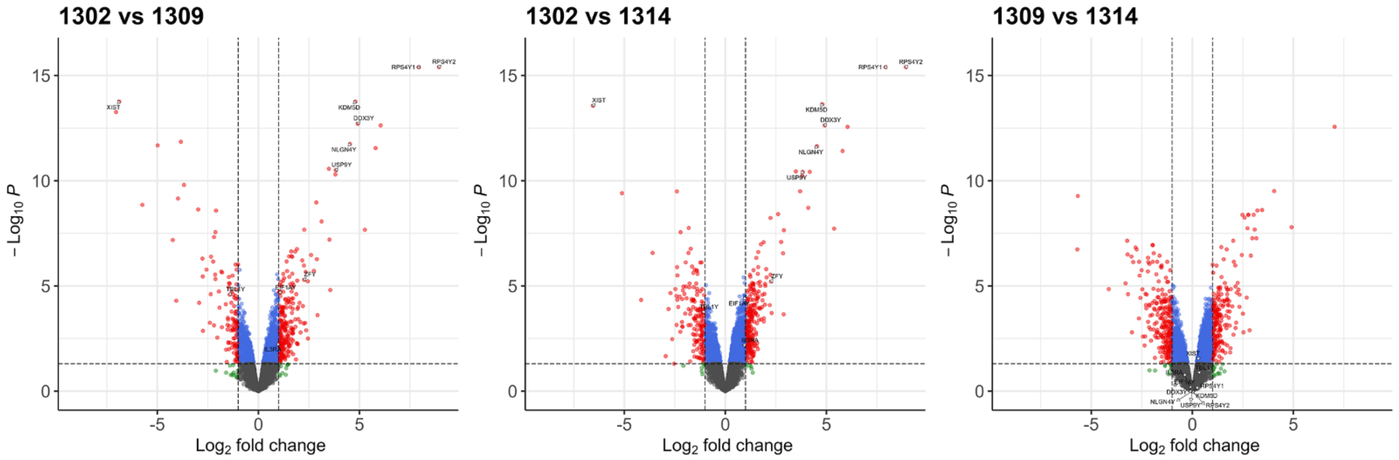

Figure S2. Visualization of gene expression differences between pairs of HUVECs on Volcano plots including the Y chromosome genes and XIST. For each comparison, the four different cell culture media served as replicates. The log2 transformed fold change (FC) values and the -log10 of the adjusted p-values (Benjamini-Hochberg method) of the genes were plotted for each comparison. Genes are color coded as follows: green dots: FC ≥ 2 and p-value > 0.05; blue dots: FC < 2 and p-value < 0.05; red dots: FC ≥ 2 and p-value < 0.05; grey dots: FC < 2 and p-value > 0.05. Only genes with FC ≥ 2 and p-value < 0.05 that are associated with the Y chromosome or XIST are labeled.

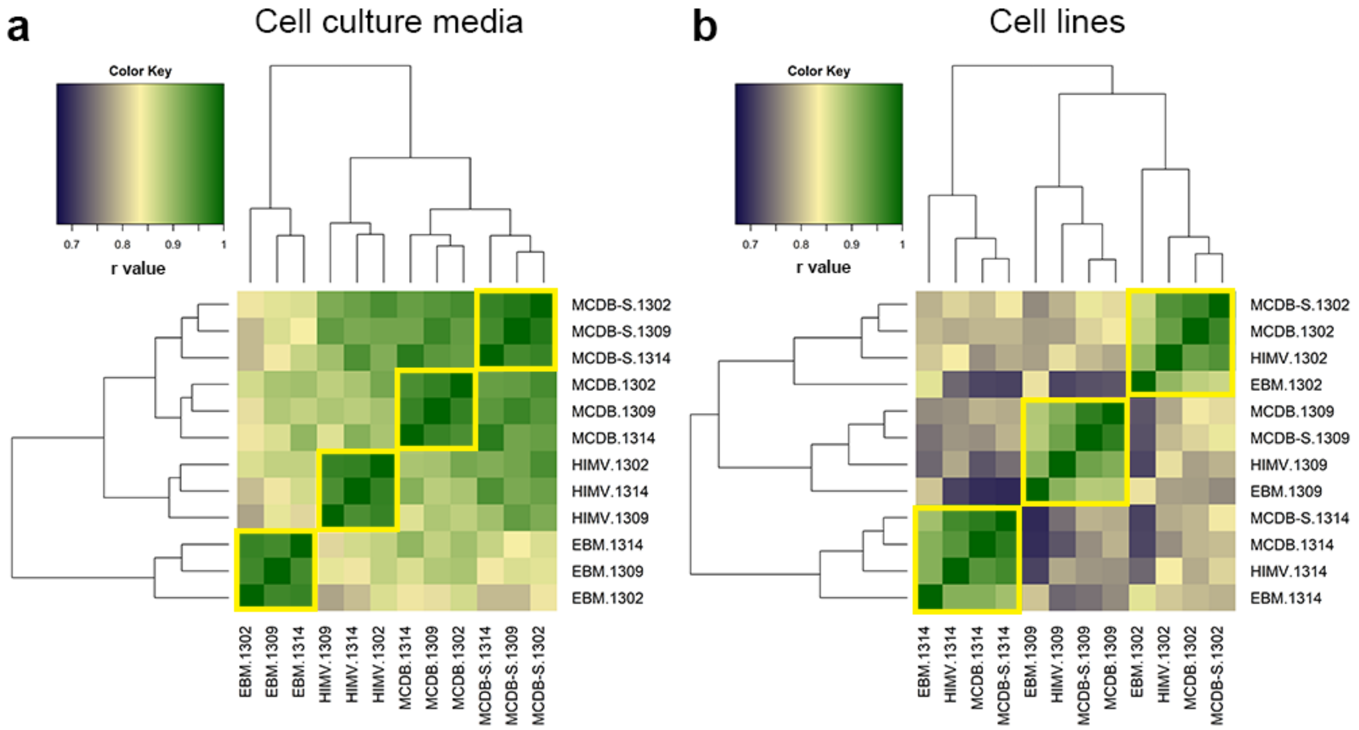

Figure S3. Correlation analysis of significantly altered genes between media (panel a) and between cell lines (panel b). The 12 samples were correlated based on the expression values of genes that exhibited a significant expression difference of at least two-fold between any two media (n = 1697, panel a) or between any two cell lines (n = 805, panel b), using Spearman correlation. The Spearman correlation coefficients (r values) are displayed using a color scale and summarized in Table S2. The dendrograms show the hierarchical clustering of the 12 samples, which was performed using the heatmap.2 function in R. The clustering was based on the Euclidean distance between the samples, with the complete linkage method applied.

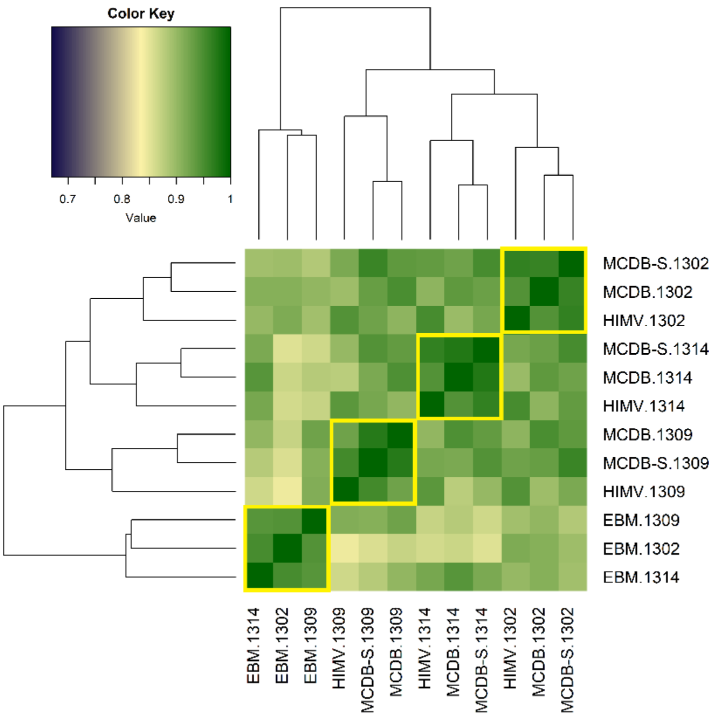

Figure S4. Correlation analysis of genes exhibiting a significant expression difference of at least 1.5-fold between any two media or any two HUVEC lines (n = 3391), using Spearman correlation. The Spearman correlation coefficients (r values) are displayed using a color scale. The dendrograms show the hierarchical clustering of the 12 samples, which was performed using the heatmap.2 function in R. The clustering was based on the Euclidean distance between the samples, with the complete linkage method applied.

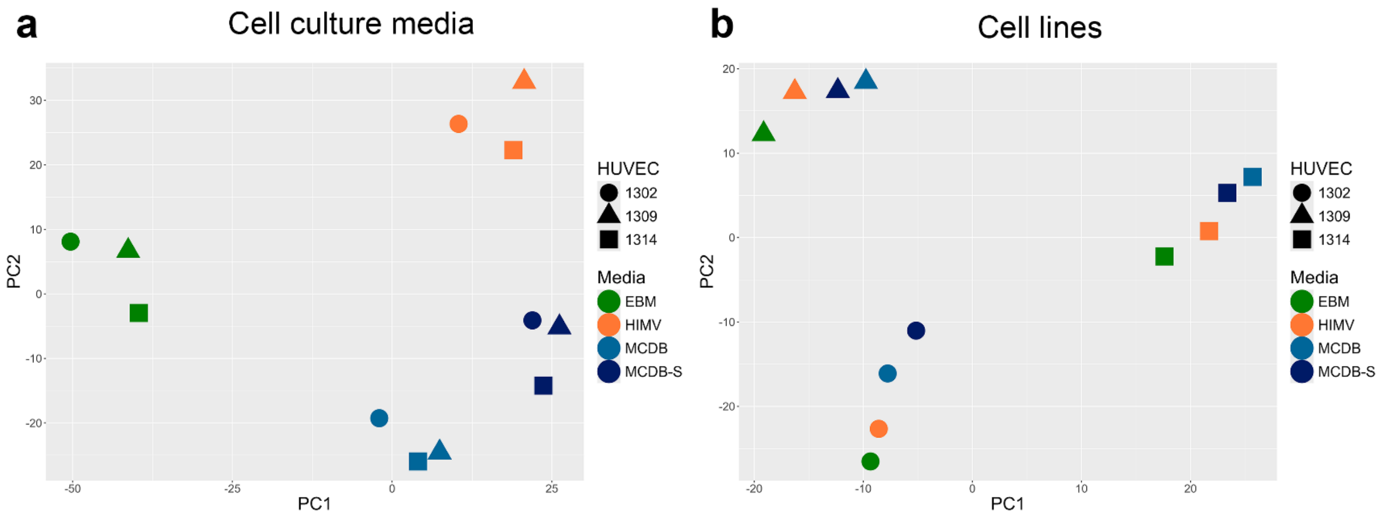

Importance of component(s):	PC1	PC2	PC3
R^2	0.4563	0.2296	0.0992
Cumulative R^2	0.4563	0.6859	0.7851

Importance of component(s):	PC1	PC2	PC3
R^2	0.3517	0.3132	0.1307
Cumulative R^2	0.3517	0.6648	0.7955

Figure S5. Principal component analysis of significantly altered genes between media (panel a) and between cell lines (panel b). Principal component analysis was performed based on z-scores of log-transformed gene expression values of genes that exhibited a significant change of at least two-fold between any two media (n = 1697, panel a) or between any two cell lines (n = 805, panel b). Three principal components (PCs) were calculated (and their R2 values were plotted), of which the first two were visualized. Different media are represented by distinct colors, while different cell lines are depicted using shapes.

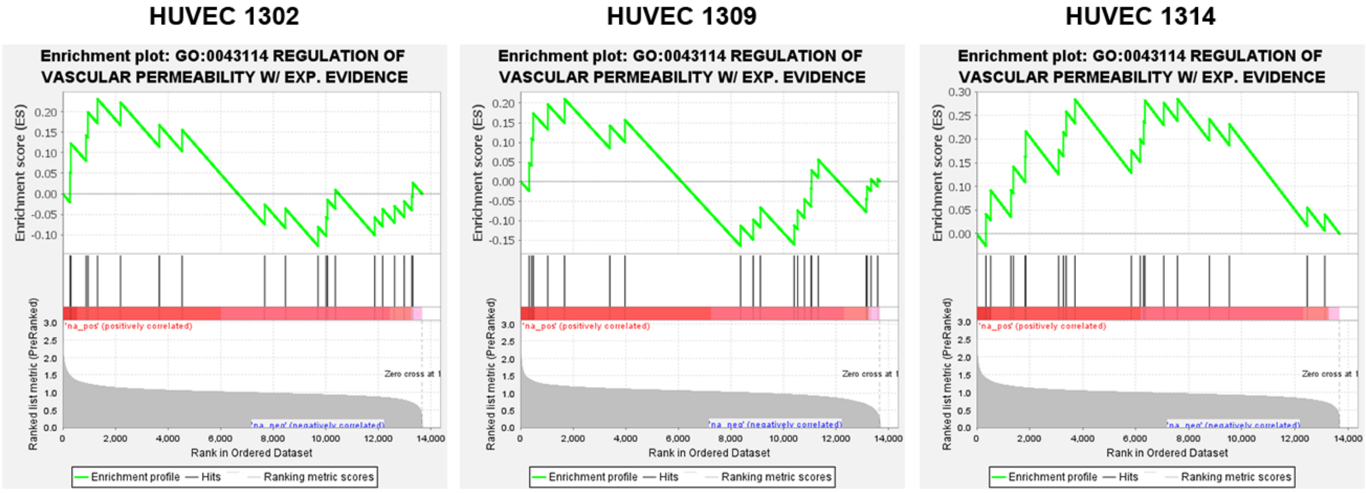

NES	p	NES	p	NES	p
1.11	0.34	1.00	0.45	1.36	0.14

Figure S6. Gene set enrichment analysis (GSEA) of the regulation of vascular permeability gene set across the three cell lines. Pre-ranked GSEA was performed using fold change values calculated by dividing the expression of each cell line in HIMV medium by the average expression of the three cell lines in the same medium. Enrichment plots, normalized enrichment scores (NESs), and nominal p values are plotted for the regulation of vascular permeability gene set. Normalized enrichment scores, colored in yellow, indicate no significant changes at the p-value threshold (0.0141) set by the Benjamini–Hochberg correction for a 5% false discovery rate.

SUPPLEMENTARY TABLES

a	Comparison of media								
	**HIMV**			**MCDB**			**MCDB-S**		
	Gene	log2 FC	adjusted P	Gene	log2 FC	adjusted P	Gene	log2 FC	adjusted P
**EBM**	NEURL3	-4.85	0.00000	UBD	-5.84	0.00001	UBD	-6.69	0.00000
	CCL2	-4.39	0.00000	NPPB	-5.45	0.00038	IGFBP5	-6.16	0.00010
	CBLN2	-4.20	0.00001	IGFBP5	-5.02	0.00074	NPPB	-5.72	0.00014
**HIMV**				NPPB	-4.34	0.00166	NPPB	-4.61	0.00202
				CYP1A1	3.47	0.00001	MMP1	4.41	0.00001
				CXCR4	3.30	0.00073	IGFBP5	-4.26	0.00306
**MCDB**							NEK2	2.07	0.00038
							DEPDC1	1.99	0.00058
							HIST1H1A	1.93	0.00110

b	Comparison of HUVEC lines					
	**1309**			**1314**		
	Gene	log2 FC	adjusted P	Gene	log2 FC	adjusted P
**1302**	FAM224 A	-5.80	1.02E-11	FAM224A	5.80	3.06E-11
	FAP	-5.74	2.76E-09	TMEM200A	5.38	3.38E-08
	TMEM20 0A	5.27	3.48E-08	LAMC2	-5.12	9.64E-10
**1309**				COL1A1	-5.69	9.20E-06
				LAMC2	-5.67	8.47E-07
				FAP	4.92	1.62E-05

Table S1. The top three genes with the highest fold change (FC) values for each pairwise comparison of media (Panel a) and HUVEC lines (Panel b). Log2-transformed FC values and adjusted p-values are provided for each gene. The sign of the FC values indicates whether expression is higher or lower in the medium (Panel a) or cell line (Panel b) listed in the rows compared to the ones in the columns. For example, the expression of NEURL3 is lower in EBM than in HIMV.

a	**Media + cell lines (n = 2215)**											
	**EBM 1302**	**EBM 1309**	**EBM 1314**	**HIMV 1302**	**HIMV 1309**	**HIMV 1314**	**MCDB 1302**	**MCDB 1309**	**MCDB 1314**	**MCDB-S 1302**	**MCDB-S 1309**	**MCDB-S 1314**
**EBM 1302**	1.0000	0.9256	0.9377	0.8839	0.7852	0.8075	0.8826	0.8208	0.8199	0.8578	0.8018	0.8011
**EBM 1309**	0.9256	1.0000	0.9220	0.8541	0.8777	0.8183	0.8711	0.8985	0.8372	0.8417	0.8782	0.8163
**EBM 1314**	0.9377	0.9220	1.0000	0.8652	0.8126	0.8896	0.8842	0.8670	0.9203	0.8569	0.8362	0.8922
**HIMV 1302**	0.8839	0.8541	0.8652	1.0000	0.9268	0.9360	0.9304	0.8734	0.8662	0.9544	0.9002	0.8933
**HIMV 1309**	0.7852	0.8777	0.8126	0.9268	1.0000	0.9160	0.8596	0.9000	0.8337	0.8919	0.9391	0.8670
**HIMV 1314**	0.8075	0.8183	0.8896	0.9360	0.9160	1.0000	0.8767	0.8656	0.9252	0.9101	0.8937	0.9548
**MCDB 1302**	0.8826	0.8711	0.8842	0.9304	0.8596	0.8767	1.0000	0.9346	0.9193	0.9583	0.9109	0.9095
**MCDB 1309**	0.8208	0.8985	0.8670	0.8734	0.9000	0.8656	0.9346	1.0000	0.9291	0.9149	0.9648	0.9116
**MCDB 1314**	0.8199	0.8372	0.9203	0.8662	0.8337	0.9252	0.9193	0.9291	1.0000	0.9041	0.8930	0.9720
**MCDB-S 1302**	0.8578	0.8417	0.8569	0.9544	0.8919	0.9101	0.9583	0.9149	0.9041	1.0000	0.9446	0.9362
**MCDB-S 1309**	0.8018	0.8782	0.8362	0.9002	0.9391	0.8937	0.9109	0.9648	0.8930	0.9446	1.0000	0.9225
**MCDB-S 1314**	0.8011	0.8163	0.8922	0.8933	0.8670	0.9548	0.9095	0.9116	0.9720	0.9362	0.9225	1.0000
**b**	**Media (n = 1697)**											
	**EBM 1302**	**EBM 1309**	**EBM 1314**	**HIMV 1302**	**HIMV 1309**	**HIMV 1314**	**MCDB 1302**	**MCDB 1309**	**MCDB 1314**	**MCDB-S 1302**	**MCDB-S 1309**	**MCDB-S 1314**
**EBM 1302**	1.0000	0.9576	0.9615	0.8560	0.7802	0.7978	0.8609	0.8201	0.8252	0.8260	0.7938	0.7949

**EBM 1309**	0.9576	1.0000	0.9541	0.8716	0.8499	0.8272	0.8888	0.8823	0.8577	0.8523	0.8563	0.8288
**EBM 1314**	0.9615	0.9541	1.0000	0.8724	0.8147	0.8622	0.8933	0.8731	0.9043	0.8578	0.8391	0.8699
**HIMV 1302**	0.8560	0.8716	0.8724	1.0000	0.9619	0.9641	0.9209	0.8914	0.8887	0.9486	0.9229	0.9148
**HIMV 1309**	0.7802	0.8499	0.8147	0.9619	1.0000	0.9505	0.8749	0.8846	0.8533	0.9167	0.9341	0.8924
**HIMV 1314**	0.7978	0.8272	0.8622	0.9641	0.9505	1.0000	0.8871	0.8774	0.9111	0.9289	0.9179	0.9481
**MCDB 1302**	0.8609	0.8888	0.8933	0.9209	0.8749	0.8871	1.0000	0.9645	0.9503	0.9534	0.9346	0.9320
**MCDB 1309**	0.8201	0.8823	0.8731	0.8914	0.8846	0.8774	0.9645	1.0000	0.9620	0.9407	0.9609	0.9391
**MCDB 1314**	0.8252	0.8577	0.9043	0.8887	0.8533	0.9111	0.9503	0.9620	1.0000	0.9308	0.9239	0.9695
**MCDB-S 1302**	0.8260	0.8523	0.8578	0.9486	0.9167	0.9289	0.9534	0.9407	0.9308	1.0000	0.9743	0.9633
**MCDB-S 1309**	0.7938	0.8563	0.8391	0.9229	0.9341	0.9179	0.9346	0.9609	0.9239	0.9743	1.0000	0.9557
**MCDB-S 1314**	0.7949	0.8288	0.8699	0.9148	0.8924	0.9481	0.9320	0.9391	0.9695	0.9633	0.9557	1.0000

**c**	**Cell lines (n = 805)**											
	**EBM 1302**	**EBM 1309**	**EBM 1314**	**HIMV 1302**	**HIMV 1309**	**HIMV 1314**	**MCDB 1302**	**MCDB 1309**	**MCDB 1314**	**MCDB-S 1302**	**MCDB-S 1309**	**MCDB-S 1314**
**EBM 1302**	1.0000	0.8228	0.8516	0.9037	0.7137	0.7349	0.8749	0.7226	0.7114	0.8680	0.7210	0.7099
**EBM 1309**	0.8228	1.0000	0.8015	0.7759	0.9071	0.7107	0.7733	0.8836	0.6945	0.7615	0.8788	0.6916
**EBM 1314**	0.8516	0.8015	1.0000	0.8042	0.7353	0.9118	0.7950	0.7621	0.9122	0.7909	0.7435	0.8939
**HIMV 1302**	0.9037	0.7759	0.8042	1.0000	0.8199	0.8307	0.9315	0.7830	0.7640	0.9446	0.7945	0.7850
**HIMV 1309**	0.7137	0.9071	0.7353	0.8199	1.0000	0.7834	0.7766	0.9138	0.7233	0.7907	0.9270	0.7417
**HIMV 1314**	0.7349	0.7107	0.9118	0.8307	0.7834	1.0000	0.7854	0.7671	0.9427	0.8121	0.7697	0.9557
**MCDB 1302**	0.8749	0.7733	0.7950	0.9315	0.7766	0.7854	1.0000	0.8274	0.7924	0.9588	0.8141	0.7934
**MCDB 1309**	0.7226	0.8836	0.7621	0.7830	0.9138	0.7671	0.8274	1.0000	0.7910	0.8188	0.9674	0.7840
**MCDB 1314**	0.7114	0.6945	0.9122	0.7640	0.7233	0.9427	0.7924	0.7910	1.0000	0.7965	0.7650	0.9708
**MCDB-S 1302**	0.8680	0.7615	0.7909	0.9446	0.7907	0.8121	0.9588	0.8188	0.7965	1.0000	0.8459	0.8285
**MCDB-S 1309**	0.7210	0.8788	0.7435	0.7945	0.9270	0.7697	0.8141	0.9674	0.7650	0.8459	1.0000	0.7911
**MCDB-S 1314**	0.7099	0.6916	0.8939	0.7850	0.7417	0.9557	0.7934	0.7840	0.9708	0.8285	0.7911	1.0000

Table S2. Spearman correlation coefficients (r values) from the correlation analysis were calculated based on genes exhibiting a significant change of at least two-fold: between any two media (panel b), between any two cell lines (panel c), or between either any two media or any two cell lines (panel a). In the table, n represents the number of genes included in each analysis.

REFERENCES

1 Megyeri, M. *et al.* Complement protease MASP-1 activates human endothelial cells: PAR4 activation is a link between complement and endothelial function. *Journal of immunology (Baltimore, Md. : 1950)* **183**, 3409-3416 (2009). https://doi.org:10.4049/jimmunol.0900879

2 Dubrovskyi, O., Birukova, A. A. & Birukov, K. G. Measurement of local permeability at subcellular level in cell models of agonist- and ventilator-induced lung injury. *Laboratory investigation; a journal of technical methods and pathology* **93**, 254-263 (2013). https://doi.org:10.1038/labinvest.2012.159

3 Debreczeni, M. L. *et al.* MASP-1 Increases Endothelial Permeability. *Frontiers in immunology* **10**, 991 (2019). https://doi.org:10.3389/fimmu.2019.00991
